# Dr. R. T. Knox

**Published:** 1898-11

**Authors:** 


### EDITORIAL DEPARTMENT.

F. E. DANIEL, M. D., Editor.
S. E. HUDSON, M. D., Managing Editor.
A. J. SMITH. M. D., Galveston, Associate Editor.

Published Monthly at Austin, Texas, by Drs. Daniel and Hudson. Subscription price $1.00 a year in advance.

Eastern Representative: John Guy Monihan, St. Paul Building, 220 Broadway, New York City.

Official organ of the West Texas Medical Association, the Houston District Medical Association, the Austin District Medical Society, the Brazos Valley Medical Association, the Galveston County Medical Society, and several others.

### Dr. R. T. Knox.

DR. ROBERT TAGGART KNOX, of Gonzales, Texas, died at his home in that city, July 22, 1898, aged 67 years.

Dr. Knox's death is a loss to the medical profession at large and a most grievous one to the community where he had for forty odd years labored unceasingly in the cause of humanity—in battling with disease and death. The unfortunate ones in life's struggle will miss his helping hand and his sustaining support and sympathy; they have indeed, been bereft of a friend true and tried.

Dr. Knox was one of the pillars of the medical profession of Texas, as well as one of its ornaments. His was a grand personality. To a splendid physique, a tall, dignified presence, was added a courtly manner that distinguished him in all crowds. He was a man too, of splendid professional attainments, and of ripe and mature judgment. He was genial, kind, generous; strong in his attachment to his friends and devoted to his profession. He was held in high esteem by all who were honored by his acquaintance, and especially by his colleagues of the State Medical Association. He held the office of First Vice-President, and filled other positions of honor and trust in that body.

Dr. Knox was a distinguished member of the Masonic order, and exemplified in his life and daily walks the highest ideal of the Christian gentleman—the true physician. Gonzales Lodge A. F. & A. M., of which he had been a member since 1857, passed resolutions on the occasion of his death, paying a high and deserved tribute to his worth.

Dr. Knox was a native of Danville, Kentucky; was raised and educated in that State. He was a graduate of Louisville Medical College, Class of 1854. To his diploma are attached the names of the immortals, Gross, Lunsford, P. Yandell Palmer and Flint. Mrs. Knox, who survives him, was Miss Catherine Blake, of Chester, South Carolina, to whom he was married in 1860.

Dr. Knox leaves a son to represent him in the medical profession, Dr. T. R. Knox of Hallettsville, Texas; and also a married son and daughter, who are living in Dakota.

Thus, one by one the old and honored landmarks of the medical profession of *ante bellum* days are passing away; are being "gathered to their rewards," let us hope in the spheres of Celestial light.

